# The multimodal psychotherapy of the anxiety disorders patients

**DOI:** 10.1192/j.eurpsy.2022.993

**Published:** 2022-09-01

**Authors:** B. Mykhaylov, O. Kudinova

**Affiliations:** 1 National University of Health Care named Shupik, Psychiatry, Kyiv, Ukraine; 2 Kharkiv Medical Academy of Postgraduate Education, Psychotherapy, Kharkiv, Ukraine

**Keywords:** Psychotherapy, Anxiety disorders, multimodal approach, episodic paroxismal disorders

## Abstract

**Introduction:**

Contemporary anxiety disorders are the main medical problem.

**Objectives:**

On the basis of complex psychopatological, pathopsychological research, were obtained reasons and conditions of formation, psychopathological structure, syndrome peculiarities, emotional disfunctions of patients on episodic paroxismal and generalized anxiety disorders and mixed anxiously depressed disorders.

**Methods:**

The basic method was a group psychotherapy with the elements of rational, positive, suggestive and family psychotherapy. In relation to disfunctions of emotional sphere, CBT was used for the phobic-depressive and anxious-depressive syndroms.

**Results:**

180 anxiety disorders patients were examined, by the stationary course of treatment.Decrease of general level of anxiety and internal anxiety was obtained for most patients. No spontaneous emergence of fear was practically observed. While active interviewing, patients stated that their former worries and fears have lost actuality and apparent emotional colouring become. Considerable reduction of symptomatic of the depressed circle also took place, patients started to feel joy and optimism.

**Conclusions:**

To correct emotional disfunction of patients with episodic paroxismal disorders, generalized anxiety disorders and mixed anxiously depressed disorders, psychotherapeutic correction system is optimal to use, which is built based on stepwise and multimodal principles.

**Disclosure:**

No significant relationships.

